# Social evolution of toxic metal bioremediation in *Pseudomonas aeruginosa*

**DOI:** 10.1098/rspb.2014.0858

**Published:** 2014-07-22

**Authors:** Siobhán O'Brien, David J. Hodgson, Angus Buckling

**Affiliations:** Department of Biosciences, University of Exeter, Penryn Campus, Penryn TR10 9FE, UK

**Keywords:** public goods, cooperation, *Pseudomonas*, bioremediation, bacterial communities

## Abstract

Bacteria are often iron-limited, and hence produce extracellular iron-scavenging siderophores. A crucial feature of siderophore production is that it can be an altruistic behaviour (individually costly but benefitting neighbouring cells), thus siderophore producers can be invaded by non-producing social ‘cheats’. Recent studies have shown that siderophores can also bind other heavy metals (such as Cu and Zn), but in this case siderophore chelation actually reduces metal uptake by bacteria. These complexes reduce heavy metal toxicity, hence siderophore production may contribute to toxic metal bioremediation. Here, we show that siderophore production in the context of bioremediation is also an altruistic trait and can be exploited by cheating phenotypes in the opportunistic pathogen *Pseudomonas aeruginosa*. Specifically, we show that in toxic copper concentrations (i) siderophore non-producers evolve *de novo* and reach high frequencies, and (ii) producing strains are fitter than isogenic non-producing strains in monoculture, and vice versa in co-culture. Moreover, we show that the evolutionary effect copper has on reducing siderophore production is greater than the reduction observed under iron-limited conditions. We discuss the relevance of these results to the evolution of siderophore production in natural communities and heavy metal bioremediation.

## 1. Introduction

Social behaviours in bacteria are commonplace, and are important both as model systems for understanding the evolution of cooperation and for playing a key role in determining virulence [1]. One well-studied example of such behaviour is the production of iron-scavenging public goods called siderophores. Siderophore production can be an altruistic behaviour (individually costly but benefitting neighbouring cells), and hence can be invaded by non-producing social ‘cheats’ [2]. The evolutionary maintenance of siderophore production can be explained by kin selection [3,4]: most neighbouring cells that benefit from siderophores are themselves siderophore producers [5]. While the primary role of siderophores is to chelate ferric iron, they can also play an important role in detoxifying heavy-metal-contaminated environments [6]. Here, we empirically investigate the importance of siderophore production as an altruistic trait in the context of bioremediation relative to iron uptake.

Siderophores can bind to a wide array of toxic metals (e.g. V^4+^, Cr ^3+^, Al ^3+^, Cu^2+^, Eu^3+^, Pb^2+^, Sn^2+^ and Tb^3+^), albeit with far less affinity than Fe^3+^ [7,8]. Many of these toxic metals are required in trace amounts for efficient metabolism, but are lethal at higher concentrations for bacteria [9]. For example, high concentrations of copper can cause oxidative stress, which can damage cellular DNA [10,11]. Unlike iron/siderophore complexes, which bind to species-specific siderophore receptors, allowing uptake of iron [8,12,13], siderophores bound to other heavy metals do not enter the cell efficiently [8]. As a result, siderophores can actually prevent heavy metals, which normally enter cells by diffusion, from being taken up, effectively detoxifying the environment for the microbial community. Moreover, detoxification may not just be a simple by-product of siderophore production in response to iron limitation, as recent work shows siderophore production in *Pseudomonas aeruginosa* is upregulated in toxic environments [14–16].

As with iron chelation, siderophore production in the context of heavy metal detoxification is likely to be an altruistic trait. Previous work has shown that siderophore-producing *P. aeruginosa* has greatly enhanced growth in metal-contaminated media relative to isogenic mutants that do not produce the primary *P. aeruginosa* siderophore, pyoverdine [16]. However, it has not been determined whether non-producers can invade populations of producers in this context. We confirmed this prediction using *P. aeruginosa* by (i) following the evolution and invasion of spontaneous non-producing mutants in initially clonal wild-type populations, and (ii) carrying out competition experiments between wild-type and an isogenic siderophore knockout mutant. We also carried out comparable experiments under non-toxic but iron-limited conditions to assess the relative importance of detoxification and iron uptake in shaping the evolution of siderophore production.

## 2. Experimental procedures

### (a) Bacterial strains and growth media

We used the *P. aeruginosa* strain PAO1 as a wild-type siderophore-producing strain, and an isogenic mutant strain with both primary and secondary siderophores, pyoverdine and pyochelin, knocked out [17].

Bacteria were cultured in Kings Medium B (KB) [18] (10 g glycerol, 20 g proteose peptone no. 3, 1.5 g K_2_HPO_4•_3H_2_O, 1.5 g MgSO_4•_7H_2_O, per litre) with the addition of either copper or iron sulfate. Iron sulfate acted as a control for the effect of sulfate and copper ions *per se*. Moreover, while Cu^2+^ stimulates the production of pyoverdine [16], Fe^2+^ inhibits it [19,20]. Consequently, siderophore production, and hence its cost, varies among our experimental treatments. Note that both metals repress siderophore fluorescence on chelation [8], meaning that bacterial fluorescence is a good proxy for free siderophore density in the medium. We identified a mildly toxic (624 µM; low strength) and highly toxic (6.17 mM; high strength) level of copper sulfate for wild-type, and prepared the same final concentrations of iron sulfate as a control. High copper treatments reduced population growth by 63.5% compared with high iron, whereas low copper reduced mean population growth by 29.25% compared with low iron. Note that the low iron treatment was not iron-limited, but a lower concentration of *supplemented* iron was added to the growth medium.

### (b) Evolution experiment and assay

We first carried out an evolution experiment to investigate whether toxicity is likely to accelerate the evolution of cheats. A single wild-type PAO1 colony was grown in KB medium at 37°C for 24 h, and approximately 10^5^ colony forming units (CFUs) inoculated into 48 microcentrifuge tubes containing 900 µl of KB medium supplemented with copper or iron sulfate to a final molarity of 624 µM or 6.17 mM (12 replicates within each of four treatments). The tubes were shaken horizontally at 180 r.p.m. at 37°C, aliquoting 1% (approx. 10^6^ CFUs) of each culture to new iron/copper-treated medium every 24 h for a total of 15 transfers (approx. 100 bacterial generations).

At transfer 15 the appearance of *de novo* mutants was monitored by aliquoting each population into standard KB medium (diluting by 10^−2^), grown for 24 h at 37°C, after which they were diluted and plated onto KB agar to assess total density and phenotype. Growing populations in KB medium (which does not require siderophore production) ensures any mutants that have evolved are obligate cheats rather than phenotypically plastic. As the amount of colony pigmentation varied considerably, we classified a colony showing any yellow/green pigmentation after 36 h as a producer; the primary siderophore of *P. aeruginosa*, pyoverdine, is yellow/green [21]. To complement this assay, the overall level of pyoverdine production in each population was assessed by a pyoverdine-specific excitation–emission assay [22,23]. One per cent of each population was transferred to iron-limited KB medium in a 96-well plate. Since siderophore production is repressed when there is an excess of Fe^2+^ [19,20], iron limitation ensures that siderophores are essential for growth and stimulates their production. Iron limitation was created by the addition of 100 μg ml^−1^ human apotransferrin and 20 mM sodium hydrogen carbonate (NaHCO_3_) to standard KB medium immediately before use. Populations were grown at 37°C for 24 h, after which fluorescence of 200 µl of culture at 460 nm, following excitation at 400 nm, was measured using a Biotek Synergy 2 spectrophotometer.

We carried out an additional experiment to ascertain how relevant toxic metals are in promoting cheat evolution, compared with the well-established evolution of cheats in iron-limited environments [5]. We repeated the evolution experiment as described above in non-toxic but iron-limited conditions (KB + 100 μg ml^−1^ human apotransferrin and 20 mM NaHCO_3_) and in standard KB media (nutrient-rich control), aliquoting 1% (approx. 10^6^ CFUs) of each culture to new medium every 24 h for a total of 33 transfers (approx. 200 bacterial generations). A pyoverdine-specific excitation–emission assay was then used to quantify the level of pyoverdine production in each population, controlling for cell density as described above.

### (c) Competition experiment and assay

Our short-term growth rate experiment consisted of 144 populations of bacteria (36 populations for each of the four treatments described above) grown in horizontally shaken 2 ml microcentrifuge tubes at 180 r.p.m., in 900 µl KB medium. Within each treatment, 12 populations were inoculated with approximately 10^5^ CFUs of wild-type, 12 with 10^5^ CFUs of mutant, and a further 12 with equal amounts of wild-type and mutant, so that the total number of CFUs in this group was also approximately 10^5^. Populations were incubated at 37°C for 24 h.

The intrinsic growth rate of wild-type and mutant strains was assessed by diluting and plating all populations onto KB agar, leaving for 24 h at 37°C, after which individual CFUs could be counted. Colony types were categorized by colour into either siderophore producers (yellow/green) or mutants (white) as before. We determined the Malthusian growth rate (*m*) for both wild-type and mutant strains as ln(final density/start density) [24]. Relative mutant fitness compared to wild-type (*W*.mutant) in co-culture was calculated as *m*(mutant)/*m*(wild-type), and in monoculture as *m*(mutant)/global mean(*m*(wild-type)).

### (d) Statistical analysis

R v. 2.15.1 [25] was employed for all statistical analyses. Experimental data and R codes are available from the Dryad repository: doi:10.5061/dryad.83t3c. We treated metal type and strength as fixed explanatory factors and relative mutant fitness (*W.*mutant) as our continuous response variable.

#### (i) Evolution experiments

We used a Kruskal–Wallis non-parametric test, with multiple comparisons (MC) among treatment groups, to investigate whether cheat frequency was affected by metal treatment after 100 generations. This test ensured our zero-inflated data did not violate the assumptions of a parametric test. A one-way analysis of variance (ANOVA) and Tukey test were employed to assess whether pyoverdine production was affected by metal treatment; these tests we also used to investigate any variation in final densities among treatments. We used a non-parametric Spearman rank order correlation to confirm the relationship between pyoverdine production and cheat frequency. Finally, we used Student's *t*-test to investigate whether iron limitation altered pyoverdine production, and Welch's *t*-test (to tolerate unequal variances) to assess how this change compared with any toxicity-mediated reduction in pyoverdine. All measures of pyoverdine were corrected for optical cell density to obtain *per capita* pyoverdine production.

#### (ii) Competition experiment

We used a factorial analysis of variance to investigate whether relative mutant fitness (*W*) was affected by metal type, growth conditions (mono versus co-culture) and metal strength, including all two-way interactions. Additionally, a Student's *t*-test was employed to assess the extent of mutant invasion in each treatment condition.

## 3. Results

### (a) Evolution experiment

Based on the fact that copper toxicity greatly upregulates siderophore production (making it more costly), we predicted toxic copper conditions would promote the evolution of non-siderophore-producing cheats, even when iron is abundant.

**Figure 1.**
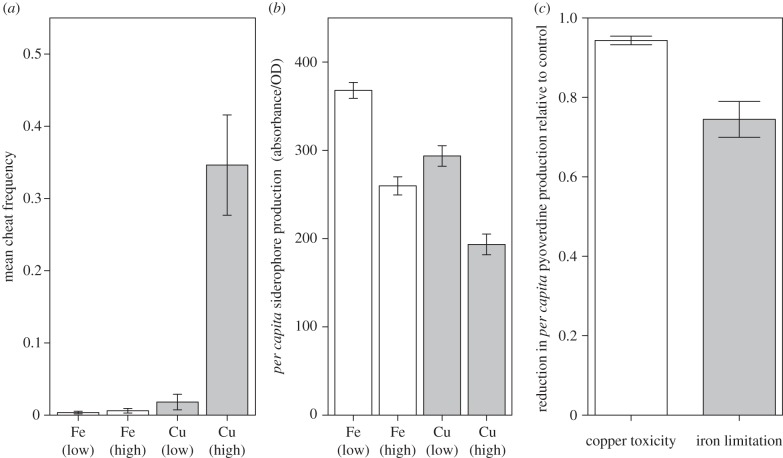
(*a*) After 100 generations, *de novo* siderophore non-producing mutants reach far higher frequencies in highly toxic copper populations than all other populations (Kruskal–Wallis and Kruskal mc: H_3_ = 29.4762, *p* < 0.001. (*b*) *Per capita* pyoverdine production is reduced after evolving in highly toxic copper for approximately 100 bacterial generations, compared with other metal treatments (one-way ANOVA and Tukey HSD, *F*_3,44_ = 45.95, *p* < 0.001). (*c*) The reduction in *per capita* pyoverdine production instigated by high copper toxicity (relative to high iron control) is stronger than the reduction caused by iron limitation (relative to standard KB broth; Welch's *t*-test, *t*_12.254_ = 4.2841, *p* < 0.005). When *y*-axis = 1, treatment has no effect of reducing pyoverdine relative to control. Error bars represent standard error.

We first carried out an evolution experiment to investigate whether heavy metal toxicity can accelerate the evolution of cheats compared with non-toxic environments. After approximately 100 generations, the proportion of cheating phenotypes was significantly higher in highly toxic (6.17 mM) copper populations than all other populations (Kruskal–Wallis and Kruskal mc: H_3_ = 29.4762, *p* < 0.001; figure 1*a*). Our assay for *per capita* pyoverdine production was consistent with this result: production was lower in highly toxic copper treatments, and higher in low iron treatments than all other treatments. (one-way ANOVA and Tukey HSD, *F*_3,44_ = 45.95, *p* < 0.001; figure 1*b*). A Spearman's rank correlation revealed a strong negative correlation between pyoverdine production and cheat frequency after 100 generations (*ρ* = −0.515, *n* = 48, *p* < 0.001). To ensure that the increased proportion of mutant phenotypes was not a result of a higher population density in the high copper treatment, we compared final population densities for each metal. Populations from high copper treatments in fact had lower densities than both iron treatments (one-way ANOVA and Tukey HSD, *F*_3,44_ = 88.36, *p* < 0.001), so density could not explain the increased proportion of mutants in high copper treatments.

To ascertain the relative importance of copper-mediated pyoverdine reduction in bacterial populations, we repeated our evolution experiment using non-toxic iron-limited and standard KB broth. After 33 daily transfers (approx. 200 bacterial generations) *per capita* pyoverdine production was lower in iron-limited populations than KB broth (Student's *t*-test, *t*_22_ = 3.6208, *p* < 0.005). Moreover, this effect of iron limitation on pyoverdine production after 200 generations was significantly weaker than the high copper-mediated decrease in pyoverdine production after just 100 generations (Welch's *t*-test, *t*_12.254_ = 4.2841, *p* < 0.005; figure 1*c*).

### (b) Competition experiment

We next carried out an ecological growth rate experiment to assess whether it is beneficial to produce siderophores in the presence of toxic copper, but costly in competition with non-producers. To do this, we measured the densities of wild-type and mutant strains grown for 24 h in monoculture and co-culture under low (624 μM) and high (6.17 mM) copper and iron conditions.

**Figure 2.**
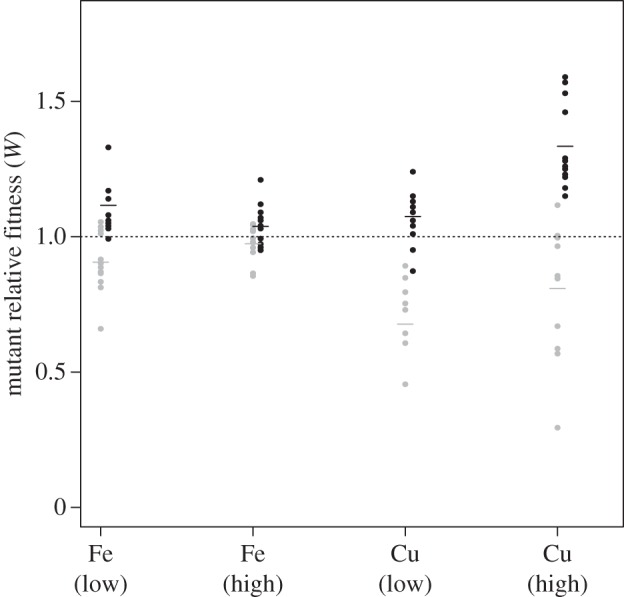
The relative fitness of mutants compared with wild-type (*W*) depends on an interaction between metal species (copper or iron) and growth conditions (monoculture, grey; co-culture, black; two-way interaction between metal and growth conditions: *F*_1,88_ = 30.583, *p* < 0.001), and an interaction between metal species and metal concentration (high = 6.17 mM, low = 624 μM; two-way interaction between metal species and concentration, *F*_1,88_ = 11.612, *p* < 0.001). In co-culture, *W* > 1 (i.e. mutants invade) for highly toxic copper concentrations (one-sample *t*-test on relative fitness, alternative = 1, *t*_11_ = 7.3307, *p* < 0.001).

We find that in the high iron treatment, mutant relative fitness is equivalent between monoculture and co-culture conditions, and relative mutant fitness is not affected by growth conditions (relative fitness = 1; figure 2). In low iron treatments, however, mutants seem to obtain a small fitness advantage in co-culture compared with monoculture. This might be explained by the cost of producing siderophores when they are not required. Moreover, when grown at both copper concentrations, mutants in monoculture have greatly reduced fitness but recoup these costs in co-culture, greatly exceeding fitness of the wild-type in high copper (figure 2; evidenced by two-way interaction between metal and growth conditions: *F*_1,88_ = 30.583, *p* < 0.001).

We also found differential impacts of metal concentrations on mutant fitness between the two metals (figure 2; evidenced by two-way interaction between metal and concentration, *F*_1,88_ = 11.612, *p* < 0.001). Whereas high copper concentration greatly increased the extent of mutant invasion compared with low copper (relative fitness more than 1; one-sample *t*-test on relative fitness, alternative = 1, high copper: *t*_11_ = 7.3307, *p* < 0.001, low copper: *t*_11_ = 2.6615, *p* < 0.05), high iron actually prevented mutants from invading, compared with low iron (one-sample *t*-test on relative fitness, alternative = 1, high iron: *t*_11_ = 1.6955, *p* = 0.118, low iron: *t*_10_ = 3.2794, *p* < 0.01). These differential impacts of metal concentration were not affected by growth conditions (evidenced by non-significance of two-way interaction between growth conditions and concentration, *F*_1,87_ = 0.0229, *p* = 0.88).

## 4. Discussion

In this study, we demonstrate that siderophore-mediated detoxification of heavy metals by bacteria is an altruistic trait, open to invasion by non-producing social cheats. We first show that spontaneous non-producing mutants in initially clonal wild-type *P. aeruginosa* populations reach higher frequencies in highly toxic compared with non-toxic environments. This is based on the premise that siderophore production is most costly when it is needed most [26], and consequently there is stronger selection for siderophore-negative mutants. We next confirm the fitness advantage of non-producing mutants in toxic (and refute it in non-toxic) environments by competing wild-type bacteria and an isogenic siderophore-negative mutant, and also demonstrate a large growth rate benefit of siderophores in toxic environments when grown as monoculture (the latter result previously shown by Braud *et al*. [16]). Finally, we validate the relative importance of toxicity-mediated social cheating by showing that the effect of toxicity in reducing pyoverdine levels is stronger than the effect of iron limitation.

A key implication of our results is that heavy metal contamination is likely to influence the evolution of siderophore production, and hence the extent of detoxification in natural populations. As for many microbial cooperative traits, precisely how will depend on population structure and the scale of decontamination [27]. If populations are highly structured, such that immediate neighbours are likely to be clonal and detoxification effects are localized, contamination may favour upregulation of siderophores through kin selection [3,4], with siderophore production carrying both direct and indirect fitness benefits. However, if populations are mixed (e.g. aquatic environments, as in this study) then there is likely to be selection for reduced siderophore production as benefits are less likely to be received by clone mates (reduced indirect benefits).

Selection for siderophore production in contaminated environments will of course also be influenced by the primary selection pressure acting on this trait: iron availability. Arguably, the ubiquity of siderophores suggests bacteria are generally iron-limited, and there is both direct and indirect evidence of siderophore exploitation by cheats in natural populations [28–33]. Toxic environments may then simply further increase selection for the production and exploitation of siderophores, in a similar response to reduced iron availability. However, there are reasons to believe that selection on siderophores for detoxification may be stronger than for iron scavenging in some contexts. First, heavy metal contamination might logically be associated with an excess of bioavailable iron. Indeed, lower pH conditions increase iron solubility (reducing the advantage of siderophores), while potentially increasing the bioavailability of toxic heavy metals. In this context, siderophore production is likely to be driven by the need for detoxification rather than iron scavenging. Second, the spatial scales of the effect of siderophores on iron acquisition and detoxification may differ, altering indirect fitness benefits. We would speculate that the spatial scale of benefit for iron acquisition is likely to be more local, simply because the binding of siderophore–iron complexes to the correct receptor facilitates the transport of iron into the cell, and hence these complexes have a shorter half-life. Third, while siderophore-mediated iron acquisition is relatively species-specific (although there is some evidence that species can exploit each other's iron–siderophore complexes [34,35]), decontamination can potentially benefit the entire community. This means that siderophore exploitation may occur not only within species but also between species. Again it is unclear how siderophore production will be affected in this context, but it is likely that this will make exploitation relatively easier, hence increasing the frequency of non-producing individuals in the populations. This multi-species interaction has recently been called the black queen hypothesis [36], and predicts that public goods will be lost first in the more abundant species and continue to be lost until any further loss is offset by the cost. Notably, our finding that toxicity-mediated pyoverdine reduction is much stronger than the reduction caused by iron limitation suggests that the social responses of *P. aeruginosa* to toxic environments may play a key role in environmental processes.

Our results may also be relevant in trying to improve the effectiveness of applying siderophore indirectly or directly for decontamination purposes. This approach has proved successful. For example, siderophores isolated from *Pseudomonas azotoformans* removed 92.8% of arsenic from the environment, much higher than EDTA (76.4%) and tap water (65.8%) [6], while the addition of *Alcaligenes eutrophus* has been demonstrated to decrease solubility of Cd, Zn and Pb in sandy soils [37]. Moreover, siderophore-producing bacteria have also been employed as a mechanism to enhance phytoremediation, whereby bacteria are added to soil containing metal-accumulating plants [38–40], although not always successfully [41]. Siderophore-containing filtrates have also been shown to enhance fungal growth, with 0.8–32.4% and 0.7–20.8% biomass increase for Cd and Zn, respectively [42].

A key point highlighted by our study is that bioremediation of any kind that relies on a steady production of siderophores may be impeded by strong selection for siderophore-negative cheats, reducing remediation efficiency. If this is the case, the implications for bioremediation are notable, whereby a high level of bacterial cooperation is essential for efficient removal of toxic materials. Notably, in natural soils, we have found copper concentrations as high as 600 ppm (E. Van-Veen 2014, personal communication), far higher than even our 392 ppm high copper treatment; therefore our results represent realistic levels of copper toxicity experienced by microbial communities. Our results predict that bioremediation may be made more efficient by establishing conditions that make cheats less viable; for example, by promoting the oxidation of Fe^2+^ ions to Fe^3+^ through the addition of lime-containing materials, or an alternative alkaline substance. Since Fe^3+^ is not readily bioavailable to bacteria, siderophores must chelate iron as well as detoxify, ensuring only populations with a high level of siderophore production are sustainable.
